# Long-Term Patient-Related Quality of Life after Knee Periprosthetic Joint Infection

**DOI:** 10.3390/jcm10050907

**Published:** 2021-02-25

**Authors:** Nike Walter, Markus Rupp, Katja Hierl, Matthias Koch, Maximilian Kerschbaum, Michael Worlicek, Volker Alt

**Affiliations:** 1 Department for Trauma Surgery, University Medical Center Regensburg, 93053 Regensburg, Germany; markus.rupp@ukr.de (M.R.); katja.hierl@ukr.de (K.H.); matthias.koch@ukr.de (M.K.); maximilian.kerschbaum@ukr.de (M.K.); michael.worlicek@ukr.de (M.W.); voker.alt@ukr.de (V.A.); 2 Department for Psychosomatic Medicine, University Medical Center Regensburg, 93053 Regensburg, Germany

**Keywords:** quality of life, periprosthetic joint infection, revision arthroplasty, psychological outcomes

## Abstract

Background: We aimed to evaluate the impact of knee periprosthetic joint infection (PJI) by assessing the patients’ long-term quality of life and explicitly their psychological wellbeing after successful treatment. Methods: Thirty-six patients with achieved eradication of infection after knee PJI were included. Quality of life was evaluated with the EQ-5D and SF-36 outcome instruments as well as with an ICD-10 based symptom rating (ISR) and compared to normative data. Results: At a follow-up of 4.9 ± 3.5 years the mean SF-36 score was 24.82 ± 10.0 regarding the physical health component and 46.16 ± 13.3 regarding the mental health component compared to German normative values of 48.36 ± 9.4 (*p* < 0.001) and 50.87 ± 8.8 (*p* = 0.003). The mean EQ-5D index reached 0.55 ± 0.33 with an EQ-5D VAS rating of 52.14 ± 19.9 compared to reference scores of 0.891 (*p* < 0.001) and 68.6 ± 1.1 (*p* < 0.001). Mean scores of the ISR revealed the psychological symptom burden on the depression scale. Conclusion: PJI patients still suffer from significantly lower quality of life compared to normative data, even years after surgically successful treatment. Future clinical studies should focus on patient-related outcome measures. Newly emerging treatment strategies, prevention methods, and interdisciplinary approaches should be implemented to improve the quality of life of PJI patients.

## 1. Introduction

Joint replacement is a life-enhancing procedure for millions of people all over the world. It provides pain relief, restores function, and preserves independence, especially in elderly patients. In Germany, primary total knee arthroplasty (TKA) is among the most common surgeries. In 2016, 168,772 TKA procedures were performed. Future numbers of TKA are expected to increase by 45% until 2040 [1]. Periprosthetic joint infection (PJI), however, is the most common complication after TKA, which puts a high burden on the patients and their families, as well as on the surgeons [2,3]. Severe limitations are often not to be avoided despite most modern and interdisciplinary treatment concepts. Patients are faced with immobility, pain, prolonged stay in hospital, multiple surgeries involving the removal of the implant and resection of the infected dead bone, as well as the administration of local and systemic antibiotics with common side effects. In general, the choice of treatment is often complex depending on multiple factors, such as duration of infection, pathogens, status, and condition of the implanted prosthesis and soft tissues. Recently, new classification systems have been proposed and attempts have been made to develop treatment algorithms in order to standardize the decision-making process for the surgeon [4,5,6,7]. Whereas a wide range of success rates in terms of infection eradication is reported [8,9,10], reimplantation is not deemed feasible in a considerable number of patients, leaving the alternatives of joint stiffening (arthrodesis) or amputation of the limb. 

While some studies provide insights into the high physical and mental burden resulting from knee PJI, little is known about the long-term impact after successful somatic treatment and achieved infection eradication. This study underlies the hypothesis that PJI patients’ quality of life is still negatively affected, even though the medical procedure is completed. Therefore, the purpose of the current study was to evaluate patients’ long-term quality of life and examine whether knee PJI patients return to a health state comparable with normative data. Well-founded data of the patient-reported impact of PJI treatment will improve clinician-patient communication concerning the outcome expectations. 

## 2. Experimental Section

Consecutive patients with periprosthetic joint infections of the knee treated in our department between September 2009 and February 2019 were retrospectively studied. There were 112 cases identified defined by the criteria of the European Bone and Joint Infection Society (EBJIS) [6]. The questionnaires were sent by post and were returned by 41 patients, yielding a response rate of 36.6%. All patients were contacted by telephone. There were seven patients (6.25%) reported to be dead. The remaining 64 patients could not be reached due to a change of phone number. Surveys with incomplete data (*n* = 5) were excluded. Hence, the final cohort included thirty-six knee PJI cases with a minimum follow-up time of one year. All patients were surgically treated and achieved infection eradication. During the time of the follow-up, no reinfection occurred and no additional surgery was required. Demographics were retrieved at the time point of the last surgery. Revision rates were defined as required surgeries between infection occurrence and infection eradication. Informed consent was obtained from all individual participants included in the study. The study was approved by the institutional ethics committee of the University Clinic of Regensburg according to the Helsinki Convention (file number 20-1681-104).

Patient-related outcome and quality of life was assessed using the German Short-Form 36 (SF-36) and EQ-5D scores as well as an ICD-10-based symptom rating (ISR). The latter is an inventory frequently used in psychosomatic anamnesis. It consists of 29 items and covers various mental syndromes with subscales for depression, anxiety, obsessive/compulsive disorders, somatoform disorders, and eating disorders [11]. The EQ-5D is a well-established generic quality of life instrument developed by the EuroQol group comprising five questions concerning the functional domains mobility, self-care, everyday life activities, pain/discomfort, and anxiety/depression [12]. The items were converted into a single EQ index value using German norm data weights [13]. Additionally, the EQ-5D was evaluated using the visual analogue scale (VAS) method [12]. The widely used SF-36 health survey captures the general health status with 36 questions in eight functional domains: physical function, role physical, bodily pain, general health, vitality, social function, emotional role, and mental health [14]. Summary scores for the physical and mental component were calculated using normative data from a German national health interview and examination survey conducted in 1998 with 7124 participants [15]. 

Data were analyzed using SPSS statistics version 24.0 (IBM, SPSS Inc., Armonk, NY, USA). Descriptive statistics were calculated for all variables. Continuous variables were expressed as the mean and standard deviation. After determining by Levene’s test that the data were normally distributed and appropriate for parametric testing, independent *t*-tests were performed for comparisons between continuous variables reported by the included patients and a reference population [15,16]. Significance was set at *p* < 0.001.

## 3. Results

In total, thirty-six patients (17 women; 19 men; mean age 71.6 (10.7) years) were included in the analysis (Table 1). The mean revision rate was 2.6 (1.9). The mean BMI reached a value of 32.8 (8.3) kg/m^2^, four patients (11.1%) were smokers and 17 patients (47.2%) reported to be former smokers. In total, nine patients had comorbid hypertension, three patients suffered from diabetes mellitus, and two patients from hypothyroidism. The mean follow-up time was 4.9 (3.5) years with a minimum of 1 year after the last surgery. There were 16 patients (44.4%) treated with the debridement with antibiotics and implant retention (DAIR) approach. A total of eight patients (22.2%) underwent a two-stage exchange and three patients (8.3%) a one-stage exchange. In nine patients (25.0%) arthrodesis was performed. 

**Figure 1:**
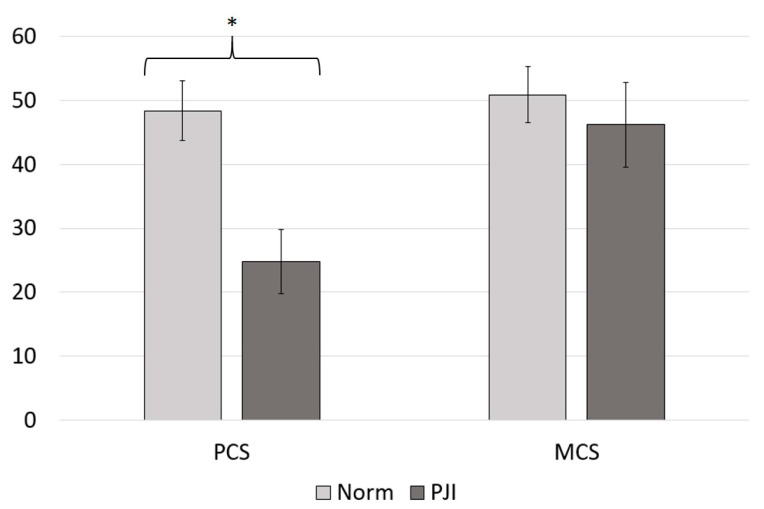
Mean physical health component score (PCS) and mean mental health component score (MCS) assessed with the SF-36. The results of the periprosthetic joint infection (PJI) cohort are shown in dark grey. For a comparison the values of the normative data are illustrated in light grey. * Significant difference.

**Figure 2:**
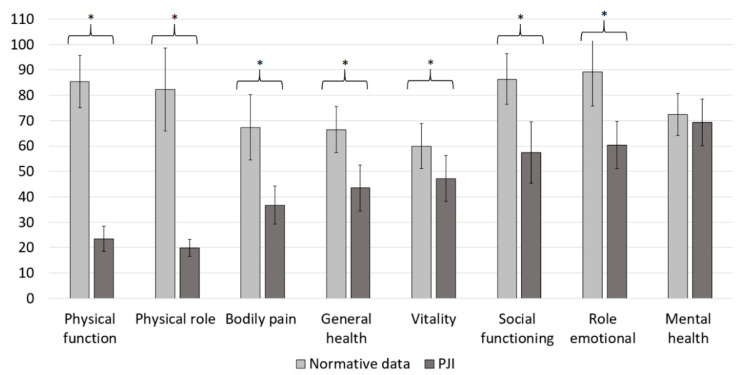
Subdimension scores for patient-related quality of life assessed with the SF-36. The results of the PJI cohort are shown in dark grey. For a comparison, the values of the normative data are illustrated in light grey. * Significant difference.

The mean physical health component score (PCS) of the SF-36 was 24.82 (10.0), and the mean mental health component score (MCS) of the SF-36 was 46.16 (13.3). In comparison with normative data from Germany, PJI patients scored lower in the physical health component (PCS_Norm_ = 48.36 (9.4), *p* < 0.001) as well as in the mental health component of the SF-36 (MCS_Norm_ = 50.87 (8.8), *p* = 0.003), which depicts 51.33% and 90.74% of the summary scores obtained from the reference population, respectively (Figure 1, Table 2) [15]. Except for the mental health dimension, PJI patients reached significantly lower values in the SF-36 subdomains (Figure 2, Table 2).

**Figure 3:**
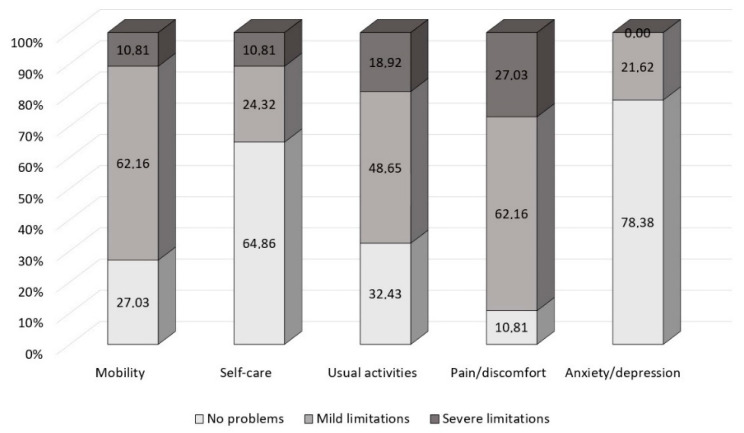
Results of the EQ-5D subdimensions given in percentage.

The mean EQ-5D index value was 0.55 (0.33), reaching 65.7% of the age-matched normative value 0.891 (*p* < 0.001) calculated based on the country-specific time trade-off value set and 61.8% of the age-matched normative value 0.838 (*p* < 0.001) calculated based on the country-specific VAS value set [16]. The mean EQ-5D VAS rating reached 52.14 (19.6), which depicts 76.0% of the score of 68.6 (1.1) obtained from of an age-matched reference population (*p* < 0.001) [16]. In the subdimensions of the EQ-5D, patients showed limited results, especially concerning pain/discomfort, mobility, and every-day life activities (Figure 3). In total, 73.0% of the patients reported any problems with mobility (compared to 15.9% of the German reference), 35.1% with self-care (compared to a norm value of 2.7%), 67.6% with usual activities (versus 9.9%), 89.2% with pain/discomfort (compared to 27.6%), and 21.6% with anxiety/depression (4.3% of the normative population). 

**Figure 4:**
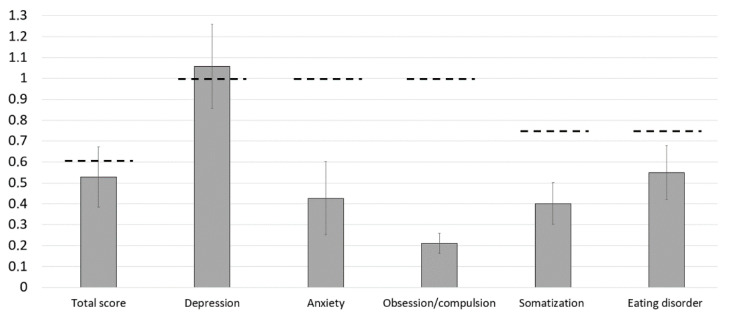
Mean values of the ISR scores. The black dotted lines depict the border of considered symptom burden.

The mean total score of the ISR was 0.53 (0.29). The mean ISR subdimension scores reached 1.06 (0.40) for depression, 0.43 (0.35) for anxiety, 0.21 (0.01) for obsessive/compulsive disorders anxiety, 0.40 (0.20) for somatoform disorders, and 0.55 (0.26) for eating disorders, respectively (Figure 4). On average, none of the values of the syndrome scales meets criteria for caseness, i.e., significant severity of psychological disorders, except for the mean score on the depression scale crossing the cut-off value of mild psychological symptom burden [11]. However, looking in more detail into the data, individuals reveal mild to severe symptom burden in the total score of the ISR (*n* = 12, 33.3%) regarding depression (*n* = 21, 58.3%), anxiety (*n* = 6, 16.7%), obsessive/compulsive disorders (*n* = 3, 8.3%), somatization (*n* = 7, 19.4%), as well as eating disorders (*n* = 8, 22.2%). 

As all the patients treated with arthrodesis were reinfection cases, a separate subgroup analysis was performed in order to determine whether outcomes are significantly worse compared to joint preserving treatment strategies. Quality of life of the nine patients (2 women; 7 men; mean age 66.4 (8.8) years) treated with arthrodesis was therefore analyzed separately at a mean follow-up time of 2.8 years, resulting in a mean EQ-5D index value of 0.48 (0.36) with a mean EQ-5D VAS rating of 41.8 (21.9). Results of the SF-36 are shown in Table 3. The mean ISR subdimension scores reached 1.33 ± 0.56 for depression, 0.31 ± 0.40 for anxiety, 0.30 ± 0.23 for obsessive/compulsive disorders anxiety, 0.37 ± 0.32 for somatoform disorders, and 0.15 ± 0.17 for eating disorders, respectively.

## 4. Discussion

In this study, the quality of life of 37 patients with successfully treated knee PJI was assessed with the questionnaires EQ-5D and SF-36. Investigating patient-reported outcomes after treatment is of clinical relevance as data illustrating the impact of PJI can improve the clinician-patient communication, especially giving insights when informing about realistic expectations of the burden of disease. The mean physical health component score of the SF-36 only reached 51.3% and the EQ-5D index value 65.7% of the normative scores, showing that even after a mean follow-up time of 4.9 years, patients who suffered from a periprosthetic infection scored significantly lower on quality of life than a German reference population. Furthermore, knee PJI patients showed reduced quality of life in comparison to patient-reported outcomes after TKA. For instance, Boonen and colleagues evaluated an EQ-5D index value of 0.90 ± 0.12 with an EQ-5D VAS rating of 76.2 ± 17.9 in 81 TKA patients at a 2-year follow up, which are 0.35 and 24.06 points higher than in our cohort [17]. The severity of PJI further becomes evident in the finding that the EQ-5D index value was even 0.13 points lower than preoperative scores obtained from 5282 patients undergoing TKA [18].

In general, studies assessing the quality of life after PJI are difficult to compare due to diverse designs. The majority evaluated specific surgical strategies, e.g., DAIR [19], arthrodesis [20] above-the-knee amputation [21] or compared the treatment choices, e.g., Winkler et al. comparing one-stage vs. two-stage exchange, Hungerer et al. comparing arthrodesis vs. above-the-knee amputation, and Preobrazhensky et al. contrasting the use of articulating vs. static spacers [22,23,24]. Cahill and colleagues compared patients with infections after total joint replacement (TRJ) with uncomplicated TRJ showing reduced SF-36 scores for the complicated group regarding the dimensions physical functioning, physical role, bodily pain, vitality, social functioning, and mental health in comparison to normative data, whereas in the uncomplicated TJR group only physical functioning was significantly decreased [25]. However, the authors did not conduct a subgroup analysis regarding the infection site or treatment option. In line with our findings are results from Helwig and colleagues, who compared the quality of life of 29 hip PJI patients with 29 knee PJI patients in the context of successful vs unsuccessful treatment including all surgical strategies. The authors report a mean PCS of 35.84 and a mean MCS of 51.73 for the knee PJI subgroup, which are significantly lower scores in the physical domain in comparison to normative data but not regarding the mental health component [26]. In contrast, in this study, no statistically significant difference was found regarding the mental health component obtained with the SF-36 in comparison to normative data, the explicit investigation of the psychological impact using the ISR revealed mild symptom burden on the depression scale on average as well as mild to severe symptom burden in the total score in a considerable proportion of the patients (32.4%). Therefore, the utilization of additional specific questionnaire capturing mental impairments might be enriching when evaluating patients’ quality of life, especially, considering that PJI is potentially life-threatening with a one-year mortality rate 4.33% and a five-year mortality rate of 21.53% [27]. Additional fears such as being dependent on external assistance, infection progression, and required medical interventions contribute to high levels of psychosocial distress [28]. Although the need for psychological support has been explicitly reported by PJI patients, no study evaluating support interventions has been conducted, thus highlighting the lack of adequate strategies in order to address the mental burden of musculoskeletal infections [29,30]. Therefore, shedding light on the psychological wellbeing of orthopedic clinical populations should be deemed as a future direction of research as an underestimation of the burden of injury may affect resource allocation, necessary prevention priorities, and may hinder the implementation of counseling as part of the standard care in trauma surgery. Additionally, routine screening for psychological comorbidities should be conducted as it has been shown that preoperatively concomitant depression or anxiety depicts a predictor of increased complications, including infection, after total joint arthroplasty as well as significant higher hospitalization charges [31,32]. To this end, the use of an ICD-10-based symptom rating can be considered an outstanding characteristic of this study. 

To further investigate the effect of recurrence of infection on patients’ quality of life, the outcomes were calculated separately for patients treated with arthrodesis. With only slightly worse scores in comparison to the whole cohort as depicted by a difference of −1.82 points in the PCS and −3.56 points in the MCS of the SF-36, knee arthrodesis can be deemed as a sufficient therapeutic alternative in cases with recurrent infections from the patients’ perspective. However, only nine patients were included in the subgroup and hence, may be underpowered.

Another key limitation of the study is the retrospective design for which reason no baseline quality of life scores for the included patients exist. Further, the findings are limited in generalizability as the sample size was relatively small with heterogeneity of the surgical procedures and no power analysis was calculated. Nonetheless, the mean follow-up time was 4.9 years, the range of follow-up time varied, and a minimum of 1 years as an inclusion criterion was short. 

Additionally, it remains questionable to what extent the lowered quality of life is attributable to the PJI and especially the SF-36 subdomain social functioning may have been influenced by other external factors, such as the current COVID-19 pandemic. As questionnaires were only administered via post, biases related to the outcomes cannot be excluded. 

In conclusion, knee PJI patients still suffer from significant lower quality of life compared to normative data, even years after surgically successful treatment. Future clinical studies should focus on patient-related outcome measures as well-founded data will be beneficial to inform patients about the impact and burden of disease and improve clinician-patient communication. 

## Figures and Tables

**Table 1:** Patient characteristics. Values are shown as mean (SD).

Patients	*n* = 36
**Gender**	
**Male**	*n* = 19 (52.8%)
**female**	*n* = 17 (47.2%)
**Age**	71.6 (10.7) years
**BMI**	32.8 (8.3) kg/m^2^
**Smoking**	
**No**	*n* = 15 (41.7%)
**Currently**	*n* = 4 (11.1%)
**Formerly**	*n* = 17 (47.2%)
**Follow-up time**	4.9 (3.5) years
**Revision rate**	2.9 (1.9)
**Surgical procedure**	
**DAIR**	*n* = 16 (44.4%)
**One-stage exchange**	*n* = 3 (8.3%)
**Two-stage exchange**	*n* = 8 (22.2%)
**Arthrodesis**	*n* = 9 (25.0%)

**Table 2:** Outcomes of the German Short-Form 36 (SF-36) in comparison to a reference population [15]. Statistical differences were determined using independent *t*-tests. Values are shown as mean (SD).

SF-36 Outcomes	All Patients (*n* = 36)	Normative Data	Independent *t*-Test
Physical health component (PCS)	24.82 (10.0)	48.36 (9.4)	*p* < 0.001
Mental health component (MCS)	46.16 (13.3)	50.87 (8.8)	*p* = 0.003
Physical function	23.4 (9.6)	85.4 (20.7)	*p <* 0.001
Physical role	19.9 (6.7)	82.36 (32.7)	*p <* 0.001
Bodily pain	36.7 (15.0)	67.38 (25.9)	*p <* 0.001
General health	43.5 (18.3)	66.42 (18.2)	*p <* 0.001
Vitality	47.2 (18.0	60.02 (17.8)	*p <* 0.001
Social functioning	57.5 (24.1)	86.36 (19.9)	*p <* 0.001
Emotional role	60.4 (18.5)	89.11 (26.7)	*p <* 0.001
Mental health	69.3 (18.5)	72.46 (16.7)	*p =* 0.003

**Table 3:** SF-36 outcomes reported from PJI patients treated with arthrodesis. Values are shown as mean (SD).

SF-36 Outcomes	Patients Treated with Arthrodesis (*n* = 9)
Physical health component (PCS)	23.0 (8.1)
Mental health component (MCS)	42.6 (23.1
Physical function	18.3 (7.3)
Physical role	5.6 (0.9
Bodily pain	35.1 (17.1)
General health	42.0 (17.1)
Vitality	43.3 (23.2)
Social functioning	51.4 (28.4)
Emotional role	45.8 (16.3
Mental health	66.4 (22.4)

## Data Availability

The data that support the findings of this study are available on request from the corresponding author, NW.
